# ST-Segment Elevation Myocardial Infarction Caused by Embolized Transcatheter Aortic Valve in Ascending Aorta

**DOI:** 10.1016/j.jaccas.2024.103125

**Published:** 2025-02-05

**Authors:** Zvonimir Ostojic, Hrvoje Jurin, Josko Bulum

**Affiliations:** a Department of Cardiovascular Diseases, University Hospital Center Zagreb, Zagreb, Croatia; b School of Medicine, University of Zagreb, Zagreb, Croatia

**Keywords:** aortic valve, complication, myocardial ischemia, stenosis, valve replacement

## Abstract

**Objective:**

Embolized valves during transcatheter aortic valve replacement can cause myocardial ischemia by obstructing diastolic retrograde blood flow.

**Key Steps:**

To prove the described mechanism of ischemia, we performed aortography with catheter above the embolized valve and placed a stiff wire through the embolized valve, causing leaflet opening while monitoring electrocardiogram. As a treatment method, an aortic stent was implanted from the embolized valve into the aortic arch, enabling backflow and resolving the ischemia.

**Potential Pitfalls:**

Overlooking the described cause of ischemia is the biggest pitfall because it can be diagnosed and treated using simple techniques.

**Take-Home Messages:**

Embolized transcatheter aortic valve replacement valve can cause myocardial ischemia by blocking the diastolic backflow of blood. Aortography and opening of the embolized valve leaflets with wire can be used for diagnosis, and aortic stent implantation in the embolized valve can be used as a treatment modality.

## Graphical Abstract

**Figure:**
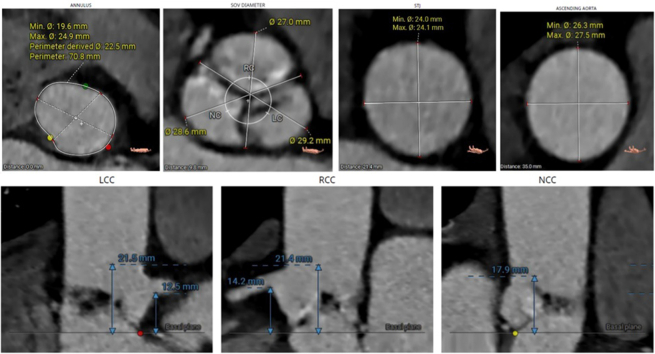

Embolized transcatheter aortic valves can cause myocardial ischemia through various mechanisms, including direct obstruction of coronary arteries and interference with diastolic flow from the aorta.^1^ Later mechanism is extremely rare, and to the best of our knowledge, it has only been described once. In that case, a patient with a large annulus and normal size of the aorta developed ischemia hours after the procedure and was diagnosed using pressure tracings.^1^

**Take-Home Messages**

• Embolized TAVR valve in the ascending aorta can cause global myocardial ischemia by blocking the diastolic backflow of blood in coronary arteries.
• Aortography with a catheter above the embolized valve and opening of the embolized valve leaflets with stiff wire can be used for diagnosis, and aortic stent implantation in the embolized valve can be used as a treatment modality.

## Case Summary

An 83-year-old female patient with symptomatic severe aortic stenosis and preserved left ventricular (LV) ejection fraction was referred for transcatheter aortic valve replacement (TAVR). The patient initially presented with cardiogenic shock due to critical aortic stenosis (maximal pressure gradient 110 mm Hg, aortic valve area 0.3 cm^2^), for which balloon aortic valvuloplasty with 20-mm balloon was performed.

**Figure 1:**
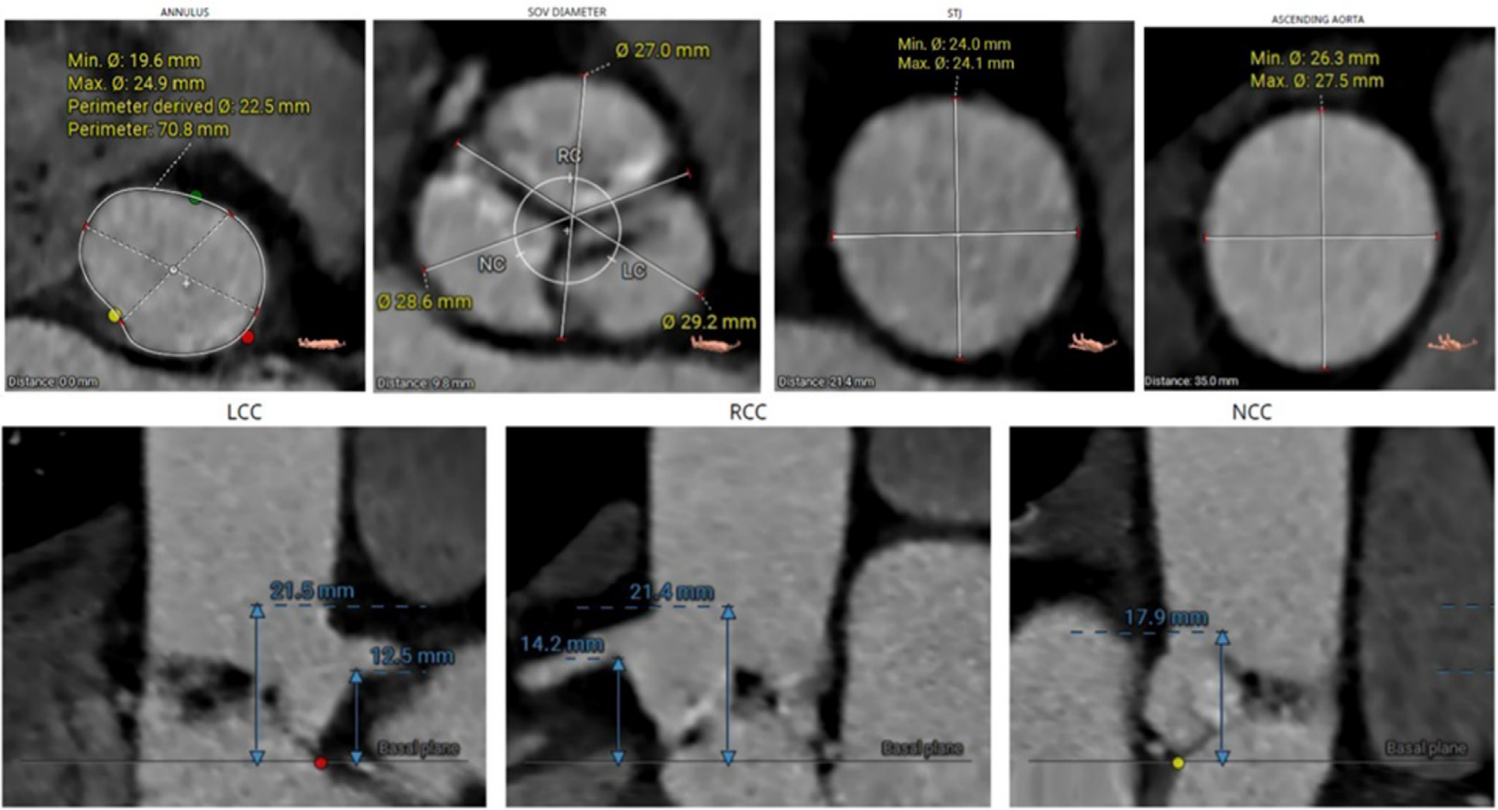
CT Measurements Obtained Prior to TAVR Computed tomography (CT) measurements obtained prior to transcatheter aortic valve replacement (TAVR) used for valve sizing. Note larger diameters of aorta compared to annulus. LC = left coronary; LCC = left coronary cusp; NC = noncoronary; NCC = noncoronary cusp; RC = right coronary; RCC = right coronary cusp; SOV = sinus of Valsalva.

**Figure 2:**
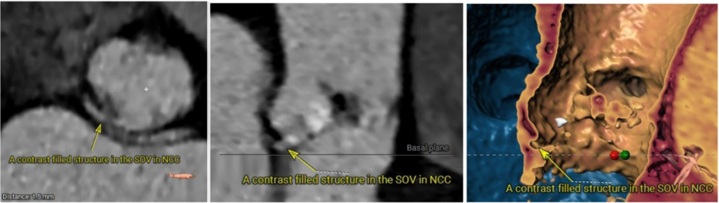
CT Representation of a Contrast-Filled Structure in the SOV of NCC A contrast-filled structure was noted in the SOV of NCC in the CT. Etiology of the finding is unknown and it could be related to prior balloon aortic valve angioplasty. Abbreviations as in Figure 1.

Computed tomography measurements are presented in Figure 1. Briefly, the annulus perimeter was 70.8 mm, coronary heights were >12 mm, and the ascending aorta diameter was 26.3 × 27.5 mm. A contrast-filled structure of unknown origin was observed in the sinus of Valsalva of the noncoronary cusp (Figure 2).

**Figure 3:**
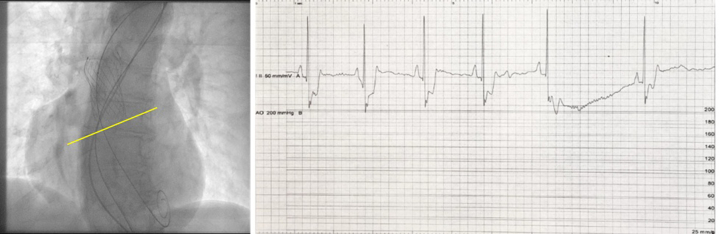
Position of the Embolized Valve in the AO and ECG Changes Following Embolization Position of the embolized valve in the aorta (AO) in relation to the aortic annulus marked with yellow line. Immediately after embolization a ST-segment denivelation and bradycardia were noted in electrocardiogram (ECG) strip, which were followed by asystole.

TAVR was performed using a self-expanding valve Evolut PRO+ 26 (Medtronic). However, the valve embolized in the aorta, causing ST-segment denivelation followed by bradycardia and asystole (Figure 3).

**Figure 4:**
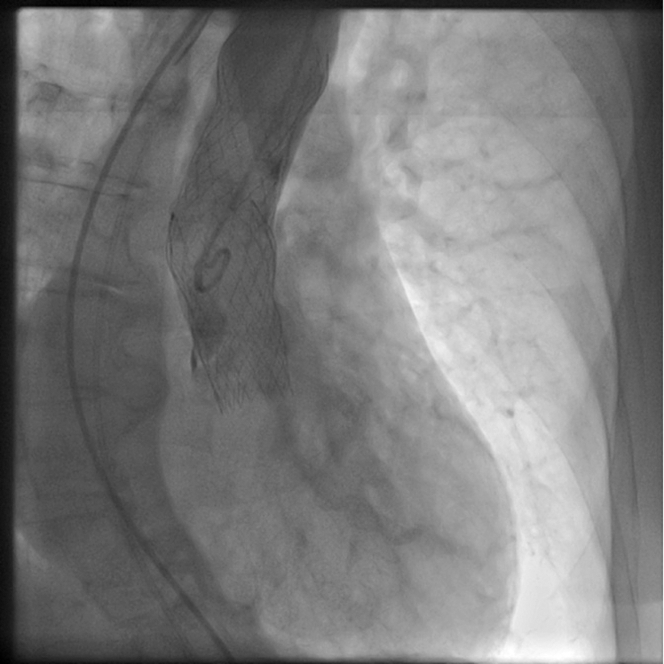
Angiographic Result After the Second Valve Implantation Angiographic result after the second valve implantation with evidence of mild paravalvular leak and patent coronary arteries.

We began cardiopulmonary resuscitation, on which asystole converted into sustained, pulseless ventricular tachycardia unresponsive to cardioversions. The patient was intubated. During the cardiopulmonary resuscitation, dilatation of the native valve was performed with a 22 × 40 mm Z-MED balloon (B Braun) (Video 1). The embolized valve was snared using One Snare 35 mm (Merit Medical) and pulled in the ascending aorta (Video 2). The second valve of the same type and size has been implanted after which successful cardioversion and hemodynamic stabilization was achieved. Aortography revealed the adequate position of the second valve with mild paravalvular regurgitation and patent coronary arteries (Figure 4, Video 3).

**Figure 5:**
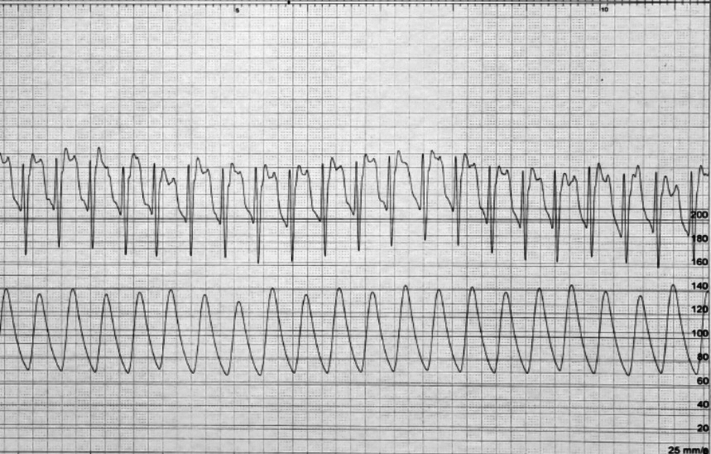
ST-Segment Elevation Noted After Catheter Removal From Embolized Valve Electrocardiogram strip demonstrating ST-segment elevation noted after catheter removal from embolized valve.

However, an ST-segment elevation developed on retrieval of the pigtail catheter in descending aorta (Figure 5).

**Figure 6:**
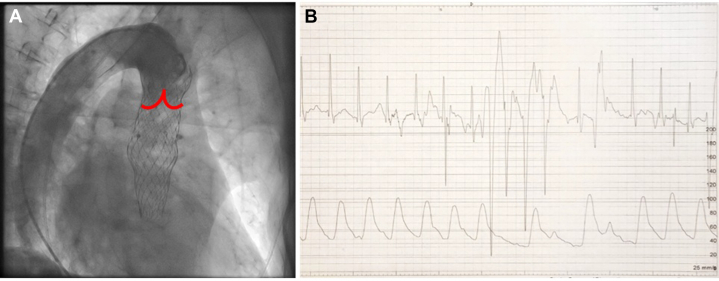
Aortography and ECG After Placement of Wire Through the Embolized Valve (A) Aortography reveals fully functional embolized valve in ascending aorta disabling retrograde flow in coronary arteries causing global myocardial ischemia. Red lines represent the position of the leaflets of the embolized valve. (B) ECG strip after placement of stiff wire through the embolized valve. ST-segment normalization occurred (as compared to Figure 5). Abbreviation as in Figure 3.

The hypothesized mechanism of the ischemia was blockage of diastolic flow from the aorta by the embolized valve, causing inadequate perfusion of coronary arteries. This was confirmed with aortography and opening of the embolized valve leaflets with Lunderquist wire (Cook Medical) (Figure 6, Video 4).

**Figure 7:**
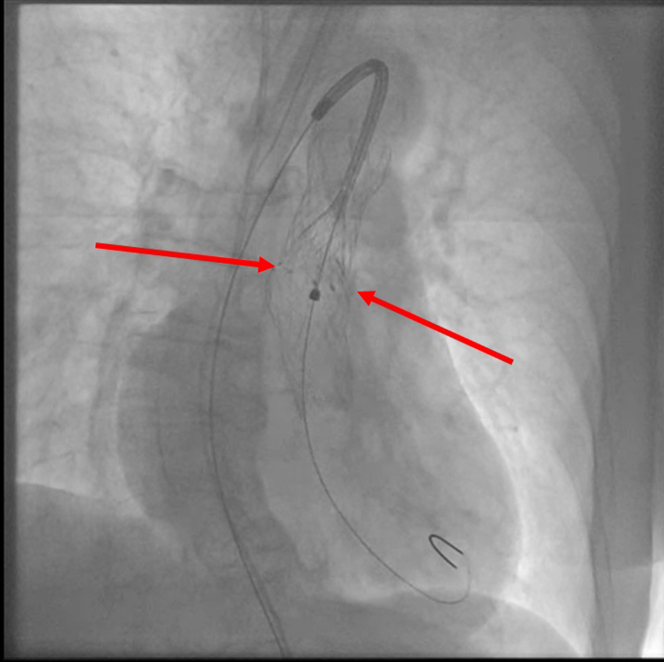
Deployment of the Aortic Stent Deployment of the aortic stent in the embolized valve. Red arrows indicate point of contact of the stent and frame of embolized valve. The position of the stent is such to fully cover leaflets of the embolized valve and not to interfere with functional valve.

An E-XL aortic stent (Jotec) 130 × 32 mm was implanted from the embolized valve into the aortic arch (Figure 7, Video 5).

**Figure 8:**
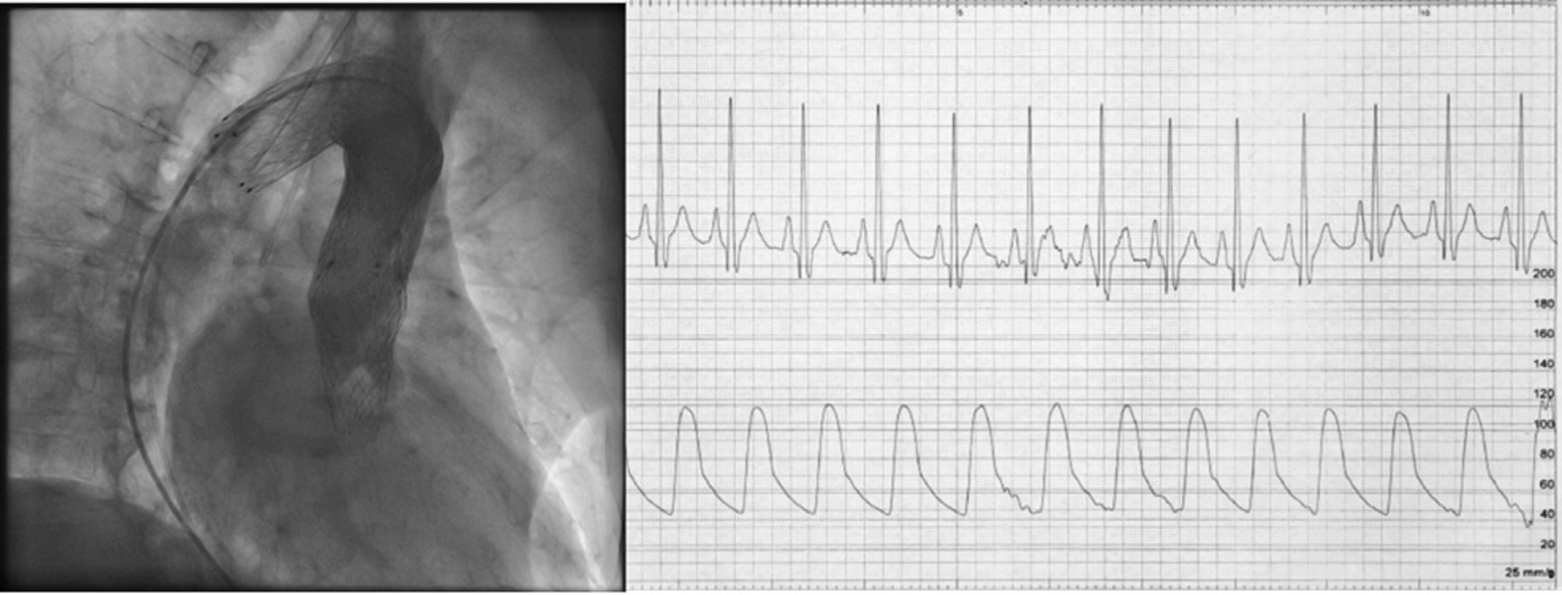
Final Aortography and ECG The final aortography revealing complete expansion of the stent, proper backflow of the blood, and patent aortic arch branches. On stent implantation, there were no ischemic changes noted in the ECG. Abbreviation as in Figure 3.

Final aortography confirmed adequate expansion of the stent, proper backflow of the blood, and patent aortic arch branches without signs of ischemia in electrocardiogram (ECG) (Figure 8, Video 6).

The patient completely recovered and was discharged on the ninth day. She has not had any cardiovascular events during the 15 months of follow-up.

## Procedural Steps

Following ultrasound vascular access acquisition, standardized techniques were used to position a Safari Small wire (Boston Scientific) in the LV. The initial valve was deployed after 1 recapture and embolized immediately on release. The precise cause of embolization has not been established. The deployment was complicated due to the tortuosity of the aorta and challenging assessment of implantation depth due to the structure in the noncoronary cusp. After the embolization, native valve dilatation was performed with a 22 × 40 mm Z-MED balloon (B Braun) (Video 1). The second valve was passed through the embolized and positioned in the native annulus. The pigtail catheter in the aortic arch was replaced by Snare One 35 mm (Merit Medical), and the embolized valve was pulled into the ascending aorta while holding the second valve in place (Video 2). Snare was exchanged with pigtail catheter, which was positioned through the embolized valve in noncoronary cups to facilitate second valve deployment, which was successfully performed. A Safari wire was removed from LV over another pigtail catheter. Control aortography revealed adequate position and function of the second valve (Video 3). The pigtail was placed within the frame of the second valve and through the embolized valve. Therefore, it kept the embolized valve leaflets open. However, on retrieval of the pigtail, an ST-segment elevation was noted.

Repeated aortography confirmed no retrograde flow below the embolized valve, the hypothesized mechanism causing ischemia (Figure 6A, Video 4). During the aortography, a 6-F pigtail catheter was placed on the embolized valve's upper border, ensuring it was not interfering with leaflets. As a second confirmation, a stiff wire (Lunderquist) was passed through the embolized valve (but not through the second valve), causing leaflet opening, after which the ST-segment normalized (Figure 6B). A pigtail catheter was used to pass the wire through the leaflets and not through the valve struts.

Lastly, an E-XL aortic stent 130 × 32 mm was implanted from the embolized valve into the aortic arch, enabling backflow (Figures 7 and 8, Videos 5 and 6). Implantation was performed over the Lunderquist wire previously placed in LV for better support. Stent was slowly and precisely released from the bottom of the embolized valve while properly covering the embolized valve leaflets and not interfering with the second (functional) valve (Video 5).

## Potential Pitfalls

When embolization occurs, an embolized valve should be pulled in the ascending aorta, especially when a coronary obstruction is a concern. If the embolized valve is Evolut, the easiest way to pull it is to snare it at 1 or both C-tab paddles. Secondary TAVR access can be used for snaring, as described in this case. We advise against removing the implantation wire from the LV because it might be challenging to get back in it before the second valve implantation. The embolized valve should not be pulled forcefully to avoid the risk of injury to the aortic wall or aortic branches.

**Figure 9:**
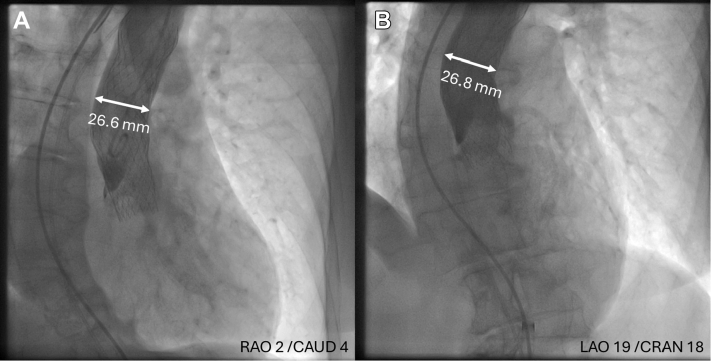
Aorta Diameter at the Inflow Part of the Embolized Valve Aorta diameter measured on aortography at the level of inflow (outer skirt) part of embolized valve from 2 projections: (A) right anterior oblique (RAO) and caudal (CAUD) and (B) left anterior oblique (LAO) and cranial (CRAN). White lines represent the position of measured aorta diameter which equals diameter of the valve (26 mm). Based on the measurements, almost complete sealing of the aorta is evident, without sufficient retrograde blood flow to maintain coronary arteries perfusion.

Not recognizing that an embolized valve can cause myocardial ischemia by blocking the aortic diastolic blood flow represents the biggest pitfall presented in this case, because most operators are unfamiliar with it due to its rarity. Indeed, observed complication has only been described once, despite relatively common valve embolizations in the same position, such as in presented case, however, primarily with older valve designs. Contemporary valves, including Evolut PRO+ used in this case, have an outer skirt that was developed to reduce paravalvular leak. The embolized valve in the described case was completely expanded, with a radius of 26 mm in the inflow (outer skirt) part that is almost the same as the size of the aorta (Figure 9). Therefore, it sealed the aorta, disabling sufficient backflow of the blood with consequent development of ischemia. Figure 9 shows aorta diameter at the inflow part of the embolized valve.

A classic aortography should be performed to confirm a diagnosis while considering that the pigtail should be above the leaflets and not interacting with them. Meticulous cine inspection should follow to make sure whether there is retrograde contrast passing through or around the embolized valve.

As a second test, we advise opening the leaflets of the embolized valve while observing changes in the ECG. This should be performed using a stiff wire with enough force to keep the leaflets open. We recommend using the Lunderquist wire because it is stiff enough, has a relatively atraumatic tip, and can be used as a working wire for stent implantation. When passing the wire through the valve, we advise using a pigtail catheter to ensure the wire does not get tangled within the valve frame. Lastly, the wire should be placed just under the embolized valve, making sure there is no interaction with the properly implanted valve.

If the ischemia mechanism is confirmed, we advise implanting the aortic stent within the embolized valve to keep the leaflets open. The stent can be implanted over the Lunderquist wire. However, before implantation of the stent, the wire should be positioned in LV over the pigtail catheter for better support. While deploying the self-expandable aortic stent, caution should be made not to interfere with the second functional valve but to cover the leaflets of the embolized valve. Therefore, initial stent release should be slow, with the target contact with the distal part of the embolized valve (Figure 7). If that is not possible due to its relation with functional valve, a higher implantation of the stent might be possible. However, one should be aware of the anatomical characteristics of the embolized valve. More precisely, the operator should know at which height from the bottom leaflets are sutured to understand at which height the stent can be implanted. In the case of the Evolut platform, which is 13 mm, it provides some space to implant the aortic stent higher in the embolized valve.

## Conclusions

Embolized TAVR valve in the ascending aorta can cause global myocardial ischemia by blocking the diastolic backflow of blood in coronary arteries. This extremely rare complication should be considered in cases with evident ischemia without an apparent cause. Furthermore, it can occur when the ascending aorta diameter is wider than the aortic annulus. Aortography with a catheter above the embolized valve and opening of the embolized valve leaflets with stiff wire can be used for diagnosis, and aortic stent implantation in the embolized valve can be used as a treatment modality.

## Funding Support and Author Disclosures

Dr Bulum has served as proctor for Medtronic, Edwards Lifesciences, and Boston Scientific. All other authors have reported that they have no relationships relevant to the contents of this paper to disclose.

## Appendix

Video 1 Dilatation of the Native Valve With 22-mm Balloon After First Valve Embolization

Video 2 Snaring and Pull Back of the Embolized Valve After Second Valve Positioning in the Annulus Both procedures were performed during cardiopulmonary resuscitation.

Video 3 Aortography After Second Valve Implantation Revealing Adequate Position and Function With Mild Paravalvular Regurgitation Opening of embolized valve leaflets is visible; however, pigtail catheter disables full closure.

Video 4 Aortography Revealing Fully Functional Embolized Valve Without Any Retrograde Flow

Video 5 Positioning and Implantation of the Aortic Stent From Bottom of the Embolized Valve Into the Aortic Arch

Video 6 Final Aortography Revealing Complete Expansion of the Stent, Proper Backflow of the Blood, and Patent Aortic Arch Branches
